# Optimizing patient's selection for prostate biopsy: A single institution experience with multi-parametric MRI and the 4Kscore test for the detection of aggressive prostate cancer

**DOI:** 10.1371/journal.pone.0201384

**Published:** 2018-08-09

**Authors:** Sanoj Punnen, Bruno Nahar, Nachiketh Soodana-Prakash, Tulay Koru-Sengul, Radka Stoyanova, Alan Pollack, Bruce Kava, Mark L. Gonzalgo, Chad R. Ritch, Dipen J. Parekh

**Affiliations:** 1 Department of Urology, University of Miami Miller School of Medicine and Sylvester Comprehensive Cancer Center, Miami, Florida, United States of America; 2 Department of Biostatistics, University of Miami Miller School of Medicine, Miami, Florida, United States of America; 3 Department of Radiation Oncology, University of Miami Miller School of Medicine and Sylvester Comprehensive Cancer Center, Miami, Florida, United States of America; University of Chicago, UNITED STATES

## Abstract

**Objectives:**

To evaluate the performance of mpMRI and the 4Kscore test together for the detection of significant prostate cancer.

Material and methods

We selected a consecutive series of men who were referred for evaluation of prostate cancer at an academic institution and underwent mpMRI and the 4Kscore test. The primary outcome was the presence of Gleason 7 or higher cancer on biopsy of the prostate. We used logistic regression and Decision Curve Analysis to report the discrimination and clinical utility of using mpMRI and the 4Kscore test for prostate cancer detection. We modeled the probability of harboring a Gleason 7 or higher prostate cancer based on the 4Kscore test and mpMRI findings. Finally, we examined various combinations and sequences of mpMRI and the 4Kscore test and assessed the impact on biopsies avoided and cancers missed.

**Results:**

Among 300 men who underwent a 4Kscore test and mpMRI, 149 (49%) underwent a biopsy. Among those, 73 (49%) had cancer, and 49 (33%) had Gleason 7 cancer. The area under the curve (AUC) for using the 4Kscore test and mpMRI together 0.82 (0.75–0.89) was superior to using the 4Kscore 0.70 (0.62–0.79) or mpMRI 0.74 (0.66–0.81) individually (p = 0.001). Similarly, decision analysis revealed the highest net benefit was achieved using both tests.

**Conclusions:**

The 4Kscore test and mpMRI results provide independent, but complementary, information that enhances the prediction of higher-grade prostate cancer and improves patient’s selection for a prostate biopsy. Prospective trials are required to confirm these findings.

## Introduction

While screening for prostate cancer may reduce prostate cancer death, it comes at the expense of significantly over diagnosing indolent cancer.[1] An overwhelming number of men will be subjected to a biopsy of the prostate before a single life can be saved from prostate cancer death.[2,1] Alternative strategies that focus on the detection of aggressive disease are desperately needed to avoid unnecessary, invasive procedures in those who are unlikely to suffer from prostate cancer.

Multi-Parametric Magnetic Resonance Imaging (mpMRI) of the prostate has emerged as an effective tool to localize cancer within the prostate, with a 45% to 87% detection rate for clinically significant disease and 63% to 98% negative predictive value.[3] The 4Kscore test is a novel blood based biomarker that was specifically designed and externally validated to accurately detect Gleason 7 or higher prostate cancer and dramatically reduce biopsies.[4] While mpMRI and the 4Kscore test are both utilized in clinical practice today for evaluation of prostate cancer, there are no reports on the impact of using these tests together. We hypothesize that both of these tests will provide independent and complementary value, and when combined will improve the detection of clinically significant disease compared to either test alone. To test this hypothesis, we assessed men who have undergone a 4Kscore test and mpMRI for evaluation of prostate cancer at the University of Miami.

## Materials and methods

We retrospectively identified a consecutive series of men who underwent mpMRI and a 4Kscore test for evaluation of prostate cancer at the University of Miami from February 2014 to May 2017. Given this was a retrospective analysis on a limited dataset, informed consent was waived by University of Miami Institutional Review Board (protocol # 20140785).

mpMRI of the prostate was performed on a 3.0-T MRI (GE Healthcare, New Jersey, USA) without endorectal coil acquiring T2WI, DWI and DCE sequences, which were interpreted by fellowship trained radiologists. Each target was classified based on the PIRADS rating scale and scored from 1–5. Since an updated version of the original PIRADS scale was released during the study period, all targets classified initially with the original PIRADS were reread as PIRADS version 2 with blinding to any additional data. Targets with PIRADS scores of 1–3 are thought to be benign or indeterminate, while targets with a PIRADS score of 4 or 5 are thought to be suspicious for prostate cancer. The 4Kscore test is a commercially available biomarker that incorporates the levels of four kallikrein proteins (total PSA, free PSA, intact PSA, and hK2) with age, digital rectal exam findings, and previous biopsy history into a well calibrated algorithm that reports the individual probability of having a Gleason 7 or higher prostate cancer on a continuous scale from 1% to 99%.

Most of the patients were referred for a biopsy due to an elevated PSA, while some were also referred due to abnormal DRE or mpMRI. The indication for the biopsy was not based on a strict protocol, but this was decided by the provider and patient. However, the patients were seen by only two providers (SP and DP), who routinely use mpMRI and the 4Kscore for evaluation of prostate cancer.

For men who were recommended to undergo biopsy of the prostate an MRI-ultrasound fusion biopsy was performed with two cores taken from each target if a suspicious mpMRI visible target was seen (PIRADS 3 or higher in either the peripheral zone or transitional zone). In addition, all men underwent a 12-core extended template biopsy. If the mpMRI was negative and a biopsy was indicated, only a 12-core extended template was performed.

We categorized mpMRI findings as positive (PIRADS 4 or 5) or negative (PIRADS 1–3). The 4Kscore test was modeled as a continuous variable, but was also categorized into low (<7.5%), intermediate (7.5%-20%), and high (>20%) scores, which are approved cut-points for the 4Kscore test.[4] Men who underwent mpMRI and the 4Kscore test for evaluation of prostate cancer were separated into those who underwent biopsy and those who did not, and basic demographic and clinical characteristics were reported.

We selected those who underwent biopsy within 6 months of mpMRI and the 4Kscore test for further analyses. Aggressive prostate cancer was defined as Gleason 7 or higher. We compared the 4Kscore between men who had a positive and negative mpMRI using the Mann-Whitney test. We fit a multivariable logistic regression model to assess the association between mpMRI, the 4Kscore, and the presence of aggressive prostate cancer on biopsy. The area under the curve (AUC) from the receiver-operating curve (ROC) was reported and compared to the models using the mpMRI or the 4Kscore test individually using the DeLong Test. We plotted the probability of aggressive prostate cancers based on mpMRI and 4Kscore findings. Finally, we used decision curve analysis to compare the clinical utility of using mpMRI and the 4Kscore test together compared to either test separately to decide on the need for a biopsy.

Finally, we compared various strategies of combining and sequencing the 4Kscore test and mpMRI and reported the impact each pathway had on biopsies avoided, and cancers missed. Finally, we categorized the 4Kscore into low, intermediate and high scores and investigated if there was any incremental benefit in adding an mpMRI for the detection of aggressive prostate cancer at each 4Kscore range.

All analyses were performed using Stata software version 14.

## Results

**Fig 1:**
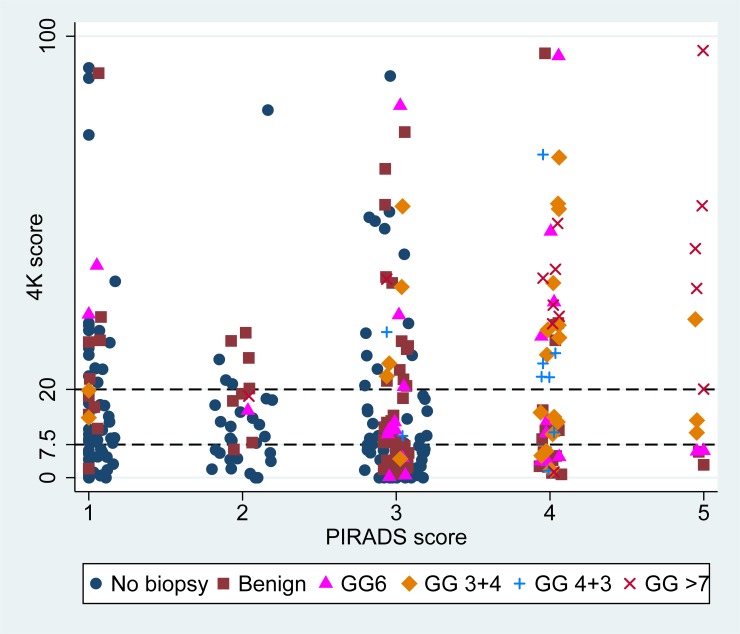
Scatter plot of the 4Kscore by PIRADS and biopsy results among 300 men who had a 4K score and a MRI for evaluation of prostate cancer.

We identified 300 consecutive men who were referred for evaluation of prostate cancer and underwent an mpMRI and 4Kscore test. 149 underwent a biopsy, allowing 151 (51%) men to avoid it. Among those, 73 (49%) had cancer, and 49 (33%) had Gleason 7 cancer. The demographic and clinical characteristics of these men are displayed in Table 1. Men who underwent a biopsy had a higher 4Kscore than those who did not [15 (6,34) vs. 7(3,16), p <0.001]. Men who underwent a biopsy were more likely to have an abnormal DRE, and a positive mpMRI. The median 4Kscore among men with a positive mpMRI was significantly higher [19 (6–39] than those with a negative mpMRI [9 (4–20)] (p = 0.0003). The distribution of 4Kscore by PIRADS and biopsy results is shown in Fig 1.

**Table 1:** Patient demographics and clinical characteristics among 300 men who underwent an MRI and 4Kscore for evaluation of prostate cancer.

	All Men with 4Kscore and mpMRI	Men with 4Kscore and mpMRI who underwent biopsy	Men with 4Kscore and mpMRI who did not undergo biopsy	p-value
	N = 300	N = 149	N = 151	
	Median (IQR)			
Age (years)	66 (60–71)	66 (61–71)	65 (60–71)	0.38
PSA (ng/ml)	6.4 (4.4–9.3)	6.3 (4.4–9.5)	6.7 (4.5–9.1)	0.13
4KScore	10 (4–27)	15 (6–34)	7 (3–16)	<0.001
	N (%)			
DRE				
Normal	247(82)	122 (49)	126 (51)	0.01
Abnormal	34 (11)	23 (68)	11(32)	
N/A	19 (7)	4 (23)	14 (77)	
Prior Biopsy				
Yes	174 (58)	82 (47)	92 (53)	0.3
No	126 (42)	67 (52)	59 (48)	
PIRADS score				
N (%)				
1	81 (27)	14(17)	67 (83)	
2	38 (13)	10 (26)	28 (74)	0.001
3	113 (35)	57 (51)	56 (49)	
4	56 (19)	56 (100)	0	
5	12 (6)	12 (100)	0	

**Fig 2:**
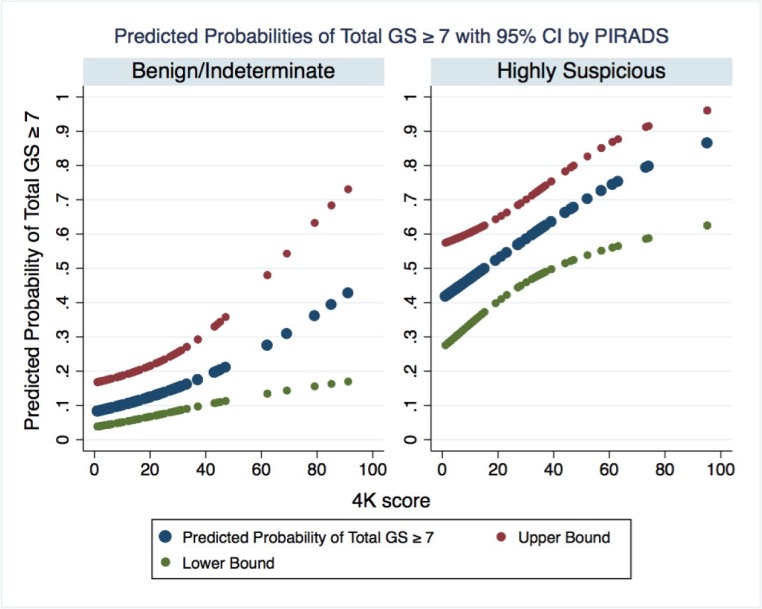
Probability of Gleason 7 cancer based on the 4Kscore and mpMRI findings among the 149 men who had mpMRI and 4Kscore and underwent biopsy of the prostate. It appears that the likelihood of Gleason 7 cancer is predicted best by the mpMRI, and the 4Kscore provides a more granular assessment of risk, within each mpMRI category.

**Fig 3:**
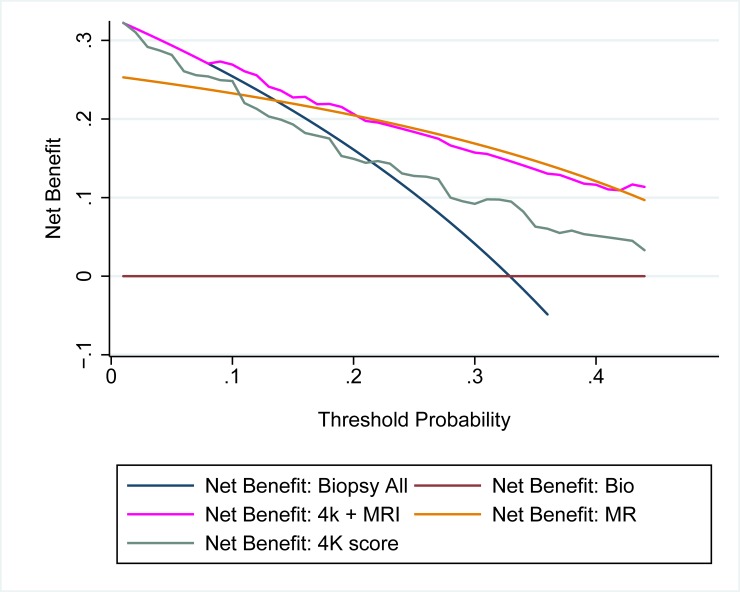
Decision curve analysis comparing clinical utility of 4Kscore, mpMRI and both 4Kscore and mpMRI for detecting clinically relevant cancer. The curve with the highest net benefit at each threshold probability is the strategy that has the best clinical utility for deciding on the need for a biopsy of the prostate.

Among the 149 men who underwent a biopsy, the AUC of using the 4Kscore test and mpMRI together to detect aggressive prostate cancer was 0.82 (0.75–0.89), compared to 0.70 (0.62–0.79) for the 4Kscore test alone and 0.74 (0.66–0.81) for mpMRI alone (p = 0.001). We found that the probability of aggressive cancer was best predicted by the mpMRI, with the 4Kscore test allowing a more precise level of risk within each mpMRI category (Fig 2). On decision analysis we found both tests together resulted in the highest net benefit compared to using either test separately to decide on the need for biopsy. While the curves for mpMRI and the 4Kscore test together and mpMRI alone appeared to overlap significantly, the clinical utility was marginally improved with both tests together at threshold probabilities between 10–20% (Fig 3).

Various strategies of combining and sequencing the 4Kscore test and mpMRI and their impact on biopsies avoided and cancer missed are presented in Table 2. If you were to defer a biopsy in men with a negative MRI and a 4Kscore < 7.5%, you would avoid 15% of the biopsies and miss only 2% of aggressive cancer. Finally, we found that a positive mpMRI was an independent predictor of aggressive cancer in the intermediate (OR 6.85, p = 0.01) and high-risk range (OR 12.5, p<0.01) of the 4Kscore, but not in the low-risk range (p = 0.08), most likely secondary to the low number of positive mpMRIs among men with a low 4Kscore.

**Table 2:** The following table reports the impact of the following 5 strategies of using the 4Kscore and/or mpMRI to determine the need for a biopsy of the prostate among 149 men who had a 4Kscore, mpMRI and biopsy of the prostate.

Strategy	Biopsies Avoided	Any cancer detected	Any cancer missed	Gleason 7+ cancer detected	Gleason 7+ cancer missed
	N (%)	N (%)	N (%)	N (%)	N (%)
	N = 149*	N = 73*	N = 73*	N = 49*	N = 49*
Strategy 1	43 (29)	59 (80)	11 (20)	43 (88)	6 (12)
Strategy 2	81 (54)	49 (67)	24 (33)	38 (77)	11 (23)
Strategy 3	124 (83)	39 (53)	34 (47)	33 (67)	16 (33)
Strategy 4	23 (15)	69 (94)	4 (6)	48 (98)	1 (2)
Strategy 5	23 (15)	69 (94)	4 (8)	48 (98)	1 (2)

*149 men underwent a prostate biopsy, of which 73 had cancer, and 49 had Gleason 7 cancer

Strategy 1: Get a 4Kscore alone and perform a biopsy for any value above 7.5%

Strategy 2: Get an mpMRI alone and perform a biopsy for a positive MRI (PIRADS 4/5)

Strategy 3: Get a 4Kscore first and if less than 7.5%, do not biopsy. If greater than 7.5%, than do mpMRI and perform a biopsy only if it is positive.

Strategy 4: Get an mpMRI first. If it is positive, then biopsy, but if negative do a 4Kscore, and only biopsy if it is above 7.5%

Strategy 5: Getting both 4Kscore and mpMRI and doing a biopsy if either 4Kscore is above 7.5% or mpMRI is positive

## Discussion

Currently, a significant number of men are subjected to a biopsy of the prostate each year for evaluation of prostate cancer.[2] These procedures are invasive, with a known risk of bleeding and life-threatening infection.[5] Furthermore, the biopsy is often negative, or reveals an indolent tumor that is unlikely to pose any threat to quantity or quality of life.[2] Consequently, more contemporary approaches to screening have focused on the detection of higher-grade disease.[6] As a result, we have seen the emergence of several non-invasive markers that have been shown to enhance prostate cancer detection.[7] Similarly, we have seen an increased utilization of mpMRI for the same purpose.[3] However, few studies have actually investigated the impact of using these tests together. Bussetto et al. assessed 171 men who had an mpMRI and PCA3 prior to undergoing a biopsy, and found that while both tests improved the detection of prostate cancer compared to standard clinical information, there was very little incremental benefit of adding a PCA3 if you already had an mpMRI.[8] Similarly, in a study of 170 men who underwent a PCA3, PHI, and mpMRI prior to repeat TRUS biopsy, there was little value in adding a biomarker after the mpMRI.[9] However, Fenstermaker et al. investigated 187 men who had a PCA3 and mpMRI prior to a MRI-US fusion biopsy and found that PCA3 improved the accuracy of prostate cancer detection if the mpMRI was negative (p<0.01), but it failed to add any benefit if the mpMRI was positive (p = 0.34).

To our knowledge this is the first study to assess the combined utility of mpMRI and the 4Kscore test for detecting aggressive prostate cancer. We found a significant number of men were able to avoid a biopsy by using these tests. Among those who underwent a biopsy, we found that an mpMRI was a statistically significant predictor of aggressive cancer, when looking at the impact of mpMRI within specific 4Kscore ranges, but not among men with a 4Kscore less than 7.5%. This may suggest that men with a 4Kscore less than 7.5% may not benefit from an mpMRI, perhaps because their risk of an aggressive tumor is low.

The discrimination for aggressive disease was better when using both tests together, compared to either test separately. The prediction of risk was primarily set by the mpMRI, with the 4Kscore test providing a more precise assessment of risk within each mpMRI category. While decision analysis showed a similar clinical utility for using both tests together compared to mpMRI alone, there was a higher net benefit to using both tests for a threshold probability of 10–20%, which is often encountered in clinical practice. To our knowledge, this is the first study to show any improvement in clinical utility when using a biomarker in addition to mpMRI. Possible explanations include the fact that we looked only at the detection of aggressive cancer; an endpoint the 4kscore was specifically calibrated for.

We looked at different sequencing strategies to combine both tests. When we looked at a strategy of doing an initial 4Kscore, followed by a mpMRI if the 4Kscore was greater than 7.5%, and a subsequent biopsy if the mpMRI was positive, we found a 83% reduction in the number of biopsies being performed. However, this resulted in an unacceptably high 33% of aggressive cancers being missed. Clearly, more work is needed to learn the best method of combining these markers to optimize biopsies avoided and cancers missed. Most importantly, we found that men with a 4Kscore less than 7.5% and a negative mpMRI had a mere 2% chance of harboring an aggressive prostate cancer. This should give men with these findings some assurance that their risk of a significant prostate cancer is small, and they can safely avoid a biopsy of the prostate.

Some limitations need to be addresses. First, the biggest limitation of this study was its retrospective design and the fact that not all men underwent a biopsy. By focusing on only those who had a biopsy we may end up with an inflated and biased estimate of each tests performance, since the tests were used to decide on the need for a biopsy. Secondly, we analyzed the performance of these tests using biopsy as the gold standard, and therefore we might have missed cancer due to known sampling errors associated with this procedure. Another limitation of this study includes the fact that a decision to perform a biopsy was not made using a standard protocol and was based instead on the clinical discretion of the treating provider. These limitations are inherent to observation research.

The study has a number of strengths that are worth pointing out. To our knowledge, this is the first study to investigate the impact of mpMRI and the 4Kscore test on the detection of aggressive cancer, rather than any cancer, as seen in other studies. Furthermore, this is the first study to investigate the combination of mpMRI and the 4Kscore; a marker specifically designed for the detection of aggressive cancer.

## Conclusion

We assessed a cohort of men who underwent an mpMRI and 4Kscore test for evaluation of prostate cancer and found that these tests improved the selection of patients for a biopsy by avoiding a significant number of unnecessary biopsies. Each test provided independent, but complementary information to improve the accuracy of predicting aggressive disease and using both tests provided a larger negative predictive value than using either test separately. However, further evaluation under the guise of a well-done prospective trial is needed to understand the best way to combine these markers to optimize their roles in selecting men for a biopsy of the prostate.

## Supporting information

S1 File Raw dataset. (XLSX)
